# Genomic Serotyping, Clinical Manifestations, and Antimicrobial Resistance of Nontyphoidal *Salmonella* Gastroenteritis in Hospitalized Children in Ho Chi Minh City, Vietnam

**DOI:** 10.1128/JCM.01465-20

**Published:** 2020-11-18

**Authors:** Vu Thuy Duong, Hao Chung The, Tran Do Hoang Nhu, Ha Thanh Tuyen, James I. Campbell, Pham Van Minh, Hoang Le Phuc, Tran Thi Hong Chau, Nguyen Minh Ngoc, Lu Lan Vi, Alison E. Mather, Stephen Baker

**Affiliations:** a The Hospital for Tropical Diseases, Wellcome Trust Major Overseas Programme, Oxford University Clinical Research Unit, Ho Chi Minh City, Vietnam; b Children’s Hospital No. 1, Ho Chi Minh City, Vietnam; c Children’s Hospital No. 2, Ho Chi Minh City, Vietnam; d The Hospital for Tropical Diseases, Ho Chi Minh City, Vietnam; e Quadram Institute Bioscience, Norwich, United Kingdom; f University of East Anglia, Norwich, United Kingdom; g Cambridge Institute of Therapeutic Immunology & Infectious Disease (CITIID), University of Cambridge, Cambridge, United Kingdom; University of Iowa College of Medicine

**Keywords:** nontyphoidal *Salmonella*, *Salmonella* serovars, genomic serotyping, pediatric diarrhea, antimicrobial resistance, multidrug resistance

## Abstract

Nontyphoidal *Salmonella* (NTS) are among the most common etiological agents of diarrheal diseases worldwide and have become the most commonly detected bacterial pathogen in children hospitalized with diarrhea in Vietnam. Aiming to better understand the epidemiology, serovar distribution, antimicrobial resistance (AMR), and clinical manifestation of NTS gastroenteritis in Vietnam, we conducted a clinical genomics investigation of NTS isolated from diarrheal children admitted to one of three tertiary hospitals in Ho Chi Minh City. Between May 2014 and April 2016, 3,166 children hospitalized with dysentery were recruited into the study; 478 (∼15%) children were found to be infected with NTS by stool culture. Molecular serotyping of the 450 generated genomes identified a diverse collection of serogroups (B, C1, C2 to C3, D1, E1, G, I, K, N, O, and Q); however, Salmonella enterica serovar Typhimurium was the most predominant serovar, accounting for 41.8% (188/450) of NTS isolates. We observed a high prevalence of AMR to first-line treatments recommended by WHO, and more than half (53.8%; 242/450) of NTS isolates were multidrug resistant (MDR; resistant to ≥3 antimicrobial classes). AMR gene detection positively correlated with phenotypic AMR testing, and resistance to empirical antimicrobials was associated with a significantly longer hospitalization (0.91 days; *P = *0.04). Our work shows that genome sequencing is a powerful epidemiological tool to characterize the serovar diversity and AMR profiles in NTS. We propose a revaluation of empirical antimicrobials for dysenteric diarrhea and endorse the use of whole-genome sequencing for sustained surveillance of NTS internationally.

## Abstract (precis)

Nontyphoidal *Salmonella* (NTS) are among the most common etiological agents of diarrheal diseases worldwide and have become the most commonly detected bacterial pathogen in children hospitalized with diarrhea in Vietnam. Aiming to better understand the epidemiology, serovar distribution, antimicrobial resistance (AMR), and clinical manifestation of NTS gastroenteritis in Vietnam, we conducted a clinical genomics investigation of NTS isolated from diarrheal children admitted to one of three tertiary hospitals in Ho Chi Minh City.

## INTRODUCTION

With an estimated 93.8 million cases (5th to 95th percentiles, 61.8 to 131.6 million) of gastroenteritis recorded globally per annum, nontyphoidal *Salmonella* (NTS) are among the most common etiological agents of diarrheal diseases worldwide. This burden disproportionally affects young children in Asia and Africa and results in ∼155,000 deaths per year (5th to 95th percentiles; 39,000 to 303,000) (1, 2).

The *Salmonellae* are a genus of Gram-negative bacteria belonging to the *Enterobacteriaceae* family. The genus is classified into two species: Salmonella enterica and Salmonella bongori, with S. enterica comprised of a further six subspecies (3). Salmonella enterica subsp. *enterica* includes >2,500 serovars which can cause a wide range of disease in humans and animals. This extensive diversity points to the ancestral origin of this subspecies, with *Salmonella* being recovered from human remnants dating back >6,500 years ago (4). Infections caused by different NTS serovars can present with differing pathology, epidemiology, clinical presentations, and antimicrobial resistance (AMR) profiles. Clinically, NTS infections are usually observed as acute gastroenteritis with the onset of fever, vomiting, abdominal cramps, and diarrhea (5). These symptoms are typically self-limiting and resolve within 5 to 7 days. Consequently, antimicrobial treatment is deemed unnecessary and is generally not recommended (6; https://www.who.int/news-room/fact-sheets/detail/salmonella-(non-typhoidal)). However, salmonellosis can also result in invasive diseases (i.e., bloodstream infection) in immunocompromised patients, and it has a high mortality rate (7, 8). Therefore, antimicrobials remain crucial for the treatment of some *Salmonella* infections, especially in high-risk patients (6, 9). Currently, the WHO guidelines for treatment of pediatric diarrhea recommend the use of low-osmolarity oral rehydration solution (ORS), zinc, and antimicrobials for all patients with bloody diarrhea, irrespective of their age (10, 11). The first drug of choice is ciprofloxacin or one of the 3-s line alternatives: pivmecillinam, azithromycin, or ceftriaxone.

Vietnam has undergone a rapid economic transition, with improved sanitation, accelerated urbanization, and changes in the food production and supply chains. This development has been followed by a shift in the key causes of bacterial enteric infections; NTS has now become the most common bacterial etiology for children hospitalized with diarrheal illnesses (12, 13). This pattern now more closely resembles the distribution of diarrheal infections in children in high-income countries (14, 15). However, despite these apparent changes in the dynamics of enteric bacteria, the epidemiology, serovar distribution, AMR, and clinical manifestation of NTS gastroenteritis have not been characterized at scale in Vietnam. The introduction of whole-genome sequencing (WGS) and analysis as a routine methodology in low- and middle-income countries (LMICs) such as Vietnam offers an opportunity for highly detailed molecular serotyping and genotyping to infer detailed epidemiological insights. In this study, we employed genomic analysis to describe some epidemiological features of the most common NTS serovars isolated from diarrheal children admitted to one of three tertiary hospitals in Ho Chi Minh City (HCMC), Vietnam.

## MATERIALS AND METHODS

### Ethics approval and consent to participate.

This study was approved by the ethics committees of participating local hospitals and the University of Oxford Tropical Research Ethics Committee (OxTREC no. 1045-13) as detailed previously (13). Written consent from parents or legal guardians of all participants was obtained prior to enrollment. Consent for publication was incorporated as a component of entrance into the study.

### Study design.

Fecal samples were collected from a prospective, observational, cross-sectional study in Children’s Hospital No. 1, Children’s Hospital No. 2, and the Hospital of Tropical Diseases in HCMC, Vietnam, from May 2014 to April 2016. Children (aged <16 years) hospitalized with diarrhea, defined as ≥3 passages of loose stools within 24 hours along with at least one loose stool containing blood and/or mucus, were recruited into the study (11). Children were not eligible if they had suspected or confirmed intussusception at the time of enrollment. Following enrollment, a short questionnaire (requesting clinical and demographic information) was completed, and a fecal sample was collected and processed within 24 hours. All enrolled patients were provided with the routine standard-of-care practices, which may have included treatment with antimicrobials.

### Microbiological culture and antimicrobial susceptibility.

Fecal specimens were inoculated onto MacConkey agar (MC; Oxoid), xylose-lysine-deoxycholate agar (XLD; Oxoid), and into selenite broth (Oxoid) and incubated at 37°C for 18 to 24 hours. Presumptive *Salmonella* was detected based on colony morphology on XLD and MC agar and confirmed using matrix-assisted laser desorption ionization–time of flight mass spectrometry (MALDI-TOF MS) (Bruker) (11).

Antimicrobial susceptibility testing was performed using the Kirby-Bauer disc diffusion method on Mueller-Hinton agar (Oxoid) for confirmed *Salmonella* isolates, interpreted using the updated CLSI guidelines (16). The tested antimicrobial agents (Oxoid) included nalidixic acid (30 μg), ciprofloxacin (5 μg), trimethoprim-sulfamethoxazole (cotrimoxazole; 1.25/23.75 μg), ceftriaxone (30 μg), ceftazidime (30 μg), ampicillin (10 μg), amoxicillin-clavulanate (20/10 μg), azithromycin (15 μg), chloramphenicol (30 μg), gentamicin (10 μg), amikacin (30 μg), and imipenem (10 μg). For *Salmonella* spp., susceptibility to aminoglycosides *in vitro* does not translate into clinical effectiveness, and thus, it was not reported (16). Multidrug resistance (MDR) was defined as nonsusceptibility to ≥1 agent in ≥3 antimicrobial categories (13).

### Whole-genome sequencing and *in silico Salmonella* serovar typing.

Total genomic DNA was extracted from retrieved, confirmed *Salmonella* specimens (*n* = 460) using the Wizard genomic DNA extraction kit (Promega, USA) and sent to the Wellcome Trust Sanger Institute (WTSI) for WGS using the Illumina HiSeq 2500 platform, generating paired-end reads (125 bp ×2) (17). Raw sequences in FASTQ format were subjected to built-in quality checking pipeline at WTSI as described previously (18) and input into Kraken (v0.10.6) for taxonomic identification by comparison to a preset database (19). We performed a *de novo* sequence assembly using Velvet v1.2.03 and VelvetOptimizer for each isolate (20), and each read set was mapped back to the corresponding assembly to improve assembly accuracy, as performed in the WTSI analysis pipeline (21). The median numbers of contigs and *N*_50_ statistic per assembly were 44 (interquartile range [IQR], 30 to 56) and 370,546 (IQR, 283,058 to 576,586), indicating that the assemblies were of sufficient quality to be used for downstream genomic analyses.

*In silico* molecular serotyping for *Salmonella* was performed for individual genome assembly using the *Salmonella In Silico* Typing Resource (SISTR) (22). The analysis was based on the multilocus sequence typing (MLST) scheme for *Salmonella*, and serovar prediction was based on identification of genetic elements coding for the O (somatic) and H (flagellar) antigens. Additionally, we performed the read-based serotyping method for *Salmonella* (SeqSero2) for all sequenced NTS (23) and compared these outputs with those generated by SISTR.

### Identification of antimicrobial resistance genes.

AMR genes were predicted from the raw sequencing reads of each isolate using Ariba (24) (version 0.4.1), which identifies AMR determinants by assembly and alignment. A manually curated input database of known resistance genes in FASTA format, taken from the Comprehensive Antibiotic Resistance Database (CARD) (McMaster University; accessed 21 March 2017), was used as the reference database. Resistance determinants were identified if they were predicted to be functional proteins (no truncations or premature stop codons) and fit the criteria of ≥ 95% nucleotide identity and ≥50% sequence length matching. The output from ARIBA was manually curated to generate a list of high-confidence hits of acquired AMR genes. Chromosomal mutations in the quinolone resistance-determining region (QRDR) were manually detected in *gyrA*, *gyrB*, *parC*, and *parE*.

### Correlation of susceptibility phenotypes and genotypes.

The presence of AMR determinants, as identified by ARIBA, indicates a nonsusceptible genotype to the corresponding antimicrobial. The phenotypic nonsusceptibility to all tested antimicrobials in our *Salmonella* collection was compiled, with intermediate phenotypes interpreted as nonsusceptible. To determine the correlation of antimicrobial susceptibility phenotypes and genotypes, we calculated the sensitivity, specificity, positive predictive value (PPV), and negative predictive value (NPV) of the presence/absence of AMR genes, using the phenotypic data as the gold standard.

### Demographic and clinical data analysis.

Clinical and demographic data were collected from all anonymized participants and processed and analyzed using Stata v11 (StataCorp, College Station, TX, USA). The growth status of participants was assessed using the WHO Global Database on Growth and Nutrition and “Prevention and Management of Obesity for Children and Adolescents” healthcare guidelines, using the macro package of Stata v11 developed by the WHO (25, 26). Hemoglobin concentration cutoff for anemia diagnosis was assessed using the recommended WHO guidelines (27). The demographic (age, sex, nutritional status, and anemia status), clinical (diarrhea type, number of diarrhea episodes per day, hospitalization duration, and outcome), and laboratory data (neutrophil count, CRP concentration) were compared among the six most abundant sequence types (STs) (*n* ≥ 20), using the Kruskal-Wallis test (continuous variables) and chi-square/Fisher’s exact tests (categorical variables). Resulting *P* values were corrected for multiple-hypothesis testing (Bonferroni correction). Data analysis and visualization were performed using R v3.6.3 (28).

### Data availability.

The raw sequence data generated from this study are available in the European Nucleotide Archive (ENA) under the project number PRJEB9121 (accession nos. ERR1764086 to ERR1764359, ERR1788605 to ERR1788707, ERR1821189 to ERR1821283, and ERR1837087 to ERR1837088).

## RESULTS

### Clinical manifestations of nontyphoidal *Salmonella* infections.

Between May 2014 and April 2016, 3,166 children hospitalized with dysentery were recruited into the study (13); 478 (∼15%) children were identified to be infected with NTS by stool culture. However, the bacteria were successfully retrieved and subjected to WGS for 460 cases. Three isolates failed during WGS due to low DNA yield. Subsequent quality control showed that an additional seven isolates were contaminated with other bacteria (Escherichia coli, *Citrobacter*, and *Pseudomonas*). Therefore, the downstream analyses were performed on 450 NTS organisms and associated metadata. More than half of these children were male (272/450; 60.3%), with the age ranging from 1 to 135 months (median, 9 months; IQR, 6.4 to 14.9 months); 22.4% (101/450) of children were <6 months of age. These children had a median of 2 days of symptoms (IQR, 2 to 4 days) before hospitalization. One-third of this population had an abnormal growth status, with 22.2% (100/450) being overweight/obese and 12.5% (56/450) being wasted/severely wasted (Table 1). Hemoglobin concentrations, according to WHO guidelines, showed that 32.9% of these children were anemic (148/450). Sixty percent (274/450) of these children were hospitalized for acute bloody diarrhea; the remainder had mucoid diarrhea without visible blood (Table 1). Profuse diarrhea was commonly recorded with an average of 10 episodes in a 24-hour period (IQR, 6 to 10 episodes). Other symptoms, including fever (294/450; 65.3%) and vomiting (190/450; 42.2%), were also frequent. Most children (except for 25 cases) were assessed not to be dehydrated. No severe sequelae or death were recorded.

**TABLE 1:** Demographic and clinical manifestations of diarrheal pediatric patients infected with nontyphoidal *Salmonella* (*n* = 450)

Characteristic(s), treatment, or outcome	Value
Sociodemographic	
Male (no. [%])	271 (60.2)
Age in months (median [IQR])	9 (6.4–14.9)
Growth (no. [%])^*a*^	
Obese/overweight/risk of overweight	100 (22.2)
Normal	271 (63.5)
Wasted/severely wasted	56 (12.5)
Clinical symptoms	
Bloody diarrhea (no. [%])	274 (60.9)
Mucoid diarrhea (no. [%])	167 (37.1)
Number of episodes per day	10 (6–10)
Dehydration (no. [%])^*b*^	25 (5.6)
Abdominal pain (no. [%])	115 (25.6)
Fever (≥37.5°C) at enrollment (no. [%])	294 (65.3)
Vomiting (no. [%])	190 (42.2)
Hematology	
Anemia (hemoglobin level = 70–109 g/liter) (no. [%])^*c*^	148 (32.9)
Neutrophil count (10^3^/μl) (median [IQR])	4.9 (3.2–7.2)
C-reactive protein (mg/liter) (median [IQR])	29 (11.0–50.0)
Treatment (no. [%])	
Low-osmolarity oral rehydration solution	418 (92.9)
IV rehydration	31 (6.9)
Antimicrobials	423 (94.0)
Fluoroquinolones (initial treatment)	299 (70.7)
Zinc	412 (91.6)
Probiotics	300 (66.7)
Outcome	
Hospital stay (days) (median [IQR])	5 (3–7)
Recovered/improved after 3 days (no. [%])^*d*^	393 (87.3)
Not improved/worse after 3 days (no. [%])	57 (12.7)

a Obese signifies weight-for-length Z score >3 SD in children aged <24 months; body mass index (BMI)-for-age Z score >3 SD in children aged ≥24 months. Overweight represents weight-for-length Z score >2 SD in children of age <24 months; BMI-for-age Z score >2 SD in children aged ≥24 months. Wasted signifies weight-for-length Z score less than −2 SD in children aged <24 months; BMI-for-age Z score less than −2 SD in children aged ≥24 months. Severely wasted signifies weight-for-length Z score less than −3 SD in children aged <24 months; BMI-for-age Z score less than −3 SD in children aged ≥24 months (30).

b All of the dehydrated cases classified as some dehydration according to Integrated Management of Childhood Illness (31).

c Hemoglobin level cutoff according to World Health Organization guidelines (18).

d Defined as “recovered” if patient had <3 passages of loose stool in the 24 hours or “improved” if patient had less episodes of diarrhea and less mucus and/or bloody in comparison to the condition of the patient at enrollment.

### The temporal distribution of *Salmonella* sequence types.

Molecular serotyping was performed on each of the 450 assembled genomes, all of which were assigned a serotype (Table 2). Serotyping results were largely consistent between the two employed methods (SISTR and SeqSero2), with mismatch in only 4 isolates (<1%). For consistency, the serotyping results presented herein were derived from the SISTR output. All but two (S. enterica subsp. *houtenae* and S. enterica subsp. *salamae*) NTS isolates were identified as S. enterica subsp. *enterica* and comprised of a diverse collection of serogroups (B, C1, C2 to C3, D1, E1, G, I, K, N, O, and Q). Accounting for 41.8% (188/450) of isolates, S. enterica serovar Typhimurium was the most predominant serovar. This serovar was comprised of three STs. More than two-thirds (133/188) of these *S*. Typhimurium were ST34, with the majority being monophasic (*n* = 120) based on the presence of only one flagellar H antigen gene copy (antigenic formula: 4,[5],12:i:−) (29). The biphasic *S*. Typhimurium (ST36, ST19, and ST34) were associated with infection in 30, 25, and 13 cases, respectively. Other common serovars included *S*. Stanley ST29 (62/450; 13.8%) and *S*. Weltevreden ST365 (34/450;7.6%). *S*. Newport (29/450; 6.4%) consisted of four different STs, including ST46 (*n* = 22), ST31 (*n* = 5), ST2366 (*n* = 1), and ST2855 (*n* = 1). Other serovars (with, at most, 10 cases) included Kentucky (ST198), Bovismorbificans, Rissen, Saintpaul, Virchow, etc. (Table 2). Furthermore, 34 serovars were detected and associated with a limited number of cases (1 to 5 cases).

**TABLE 2:** Nontyphoidal *Salmonella* predicted serovars isolated from children with diarrheal diseases in three tertiary hospitals in Ho Chi Minh City (*n* = 450)^*a*^

O-antigen group	Serovar	No. of STs	No. of patients
O:4 (B)	Typhimurium (4,[5],12:i:–)^*b*^	34	120
	Typhimurium (4:i:1,2)	36	30
		19	25
		34	13
	Stanley (4:d:1,2)	29	62
		2,615	1
	Paratyphi B var. Java (4:b:–)	423	7
		135	1
	Sandiego (4:e,h:e,n,z15)	20	1
	Agona (4:f,g,s:–)	13	4
	Indiana (4:z:1,7)	17	2
	Chester (4:e,h:e,n,x)	343	1
		2,063	1
	Schleissheim (4:b:–)	1,578	1
		2,397	1
	Saintpaul (4:e,h:1,2)	50	6
		27	2
O:7 (C_1_)	Rissen (7:f,g:–)	469	9
	Virchow (7:r:1,2)	359	5
		197	1
	Bareilly (7:y:1,5)	203	4
		909	1
	Thompson (7:k:1,5)	26	1
		2,417	1
	Ohio (7:b:l,w)	329	1
	Mbandaka (7:z10:e,n,z15)	1,602	1
O:8 (C_2_-C_3_)	Newport (8:e,h:1,2)	46	22
		31	5
		2,366	1
		2,855	1
	Kentucky (8:i:z6)	198	10
	Bovismorbificans (8:r:1,5)	1,499	9
	Corvallis (8:z4,z23:–)	1,541	4
	Albany (8:z4,z24:–)	292	2
	Hadar (8:z10:e,n,x)	33	1
	Emek (8:g,m,s:–)	76	1
	Litchfield (8:l,v:1,2)	214	1
	Muenchen (8:d:1,2)	2,424	1
	Brunei (8:y:1,5)	2,809	1
O:9 (D_1_)	Enteritidis (9:g,m:–)	11	13
		74	4
	Panama (9:l,v:1,5)	48	1
	Javiana (9:l,z28:1,5)	2,494	3
		1,547	4
	Dublin (9:g,p:–)	74	1
O:3,10 (E_1_)	Weltevreden (3,10:r:z6)	365	34
	London (3,10:l,v:1,6)	155	5
	Give (3,10:l,v:1,7)	516	5
	Anatum (3,10:e,h:1,6)	64	2
	Lexington (3,10:z10:1,5)	1,542	1
	Meleagridis (3,10:e,h:l,w)	3,248	1
Other (G, I, K, N, O, Q)	Kedougou (13:i:l,w)	1,543	3
	Agbeni (13:g,m:–)	2,606	1
	Hvittingfoss (16:b:e,n,x)	446	2
	Orientalis (16:k:e,n,z15)	558	1
	Cerro (18:z4,z23:–)	367	1
	Johannesburg (40:b:e,n,x)	512	1
	Alachua (35:z4,z23:–)	1,298	1
	Wandsworth (39:b:1,2)	1,498	3
	Subsp. *houtenae* (43:z4,z23:–)	958	1
	Subsp. *salamae* (48:d:z6)	3,200	1

a Antigenic formulas are given in parentheses.

b Monophasic.

**FIG 1:**
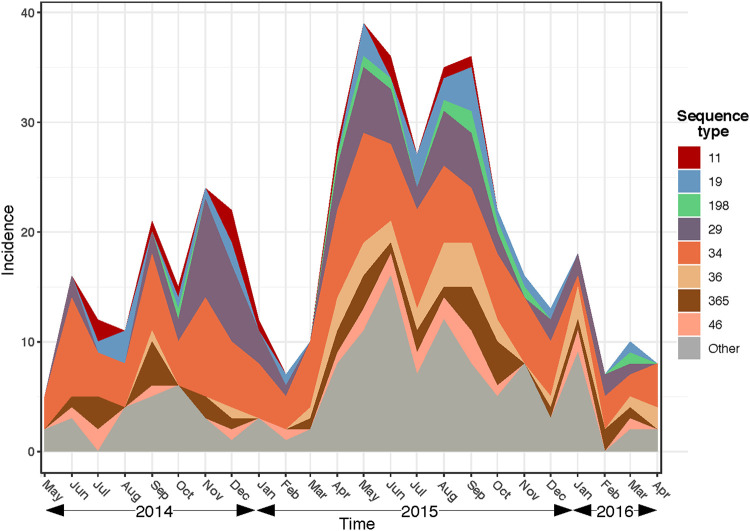
Monthly hospitalization incidence of nontyphoidal *Salmonella*. Only the eight most common sequence types are visualized. Sequence types that caused fewer than 10 incidences are grouped together as “Other.”

The eight most common STs (≥10 cases) accounted for ∼73% of all NTS and were comprised of ST34, ST29, ST36, ST365, ST19, ST46, ST11, and ST198. The temporal distribution of the diarrheal cases caused by these eight STs is shown in Fig. 1. In 2015, the absolute number of NTS cases increased from March and peaked in May and September, followed by a rapid decline. This pattern coincides with the duration of the rainy season in Southern Vietnam (from April to October). Over time, there was no apparent change in the proportional distribution of NTS genotypes, with *S*. Typhimurium (ST34) dominating the epidemiological landscape during our investigational period. The eight most common STs could be detected throughout the study, with *S*. Enteritidis (ST11) and *S*. Kentucky (ST198) more apparent during periods with more cases.

### High concordance between antimicrobial resistance genotype and phenotype.

The majority of NTS were susceptible to imipenem (445/450) and amikacin (447/450), whereas over half of the collection exhibited resistance to ampicillin and chloramphenicol. Resistance to ciprofloxacin was comparatively low (39/450), but 221 isolates exhibited reduced susceptibility to this antimicrobial. Correspondingly, ∼58% of the NTS were nonsusceptible to ciprofloxacin. Resistance against trimethoprim-sulfamethoxazole (38%), third-generation cephalosporins (13%), and azithromycin (18%) were comparatively low. Consistent with a high prevalence of resistance, more than half (53.8%; 242/450) of NTS isolates were determined to be MDR.

We exploited *in silico* methods to detect the AMR determinants (acquired genes and mutations) in all the NTS genomes. The prevalence of all detected AMR genes, along with their corresponding antimicrobial classes, is shown in Table S1 in the supplemental material. Subsequently, we assessed how detected AMR genes (classified by antimicrobial class) could predict phenotypic resistance (Table 3).

**TABLE 3:** The performance of whole-genome sequencing in determining the antimicrobial susceptibility of nontyphoidal *Salmonella*

Antimicrobial(s)	Antimicrobial resistance gene(s)	Gene(s) detected?	No. of patients with phenotype:		Sensitivity (% [95% CI])	Specificity (% [95% CI])	PPV	NPV
			Nonsusceptible^*a*^	Susceptible				
Ampicillin	*bla*_TEM-95_, *bla*_OXA-1_, *bla*_CARB-3_, *bla*_SHV-66_, *bla*_CMY_, *la*_CTX-M-55_, *bla*_CTX-M-14_/*bla*_CTX-M-15_	Yes	279	1	97.2 (94.6–98.8)	99.4 (96.6–100)	99.6	95.3
		No	8	162				
Ceftriaxone/ceftazidime	*bla*_CTX-M-55_, *bla*_CTX-M-14_, *bla*_CTX-M-15_	Yes	52	3	91.2 (80.7–97.1)	99.2 (97.8–99.8)	94.5	98.7
		No	5	390				
Imipenem	*bla*_NDM-1_	Yes	0	1	0.0 (0.0–52.2)	99.8 (98.8–100)	0.0	98.9
		No	5	444				
Gentamicin	*aac(3)-IIa*, *aac(3)-IV*, *aac(6′)-IIa*	Yes	89	2	100 (95.9–100)	99.4 (98.0–99.9)	97.8	100
		No	0	358				
Azithromycin	*mphA*, *mefB*, *ermT*, *ermF*, *ermB*	Yes	59	7	72.8 (61.8–82.1)	98.1 (96.1–99.2)	89.4	94.3
		No	22	362				
Ciprofloxacin	QRDR^*b*^ mutation and/or PMQR^*c*^ genes	Yes	236	13	90.8 (86.8–94.0)	93.2 (88.6–96.3)	94.8	88.1
		No	24	177				
Nalidixic acid	QRDR mutation and/or PMQR genes	Yes	179	70	96.2 (92.4–98.5)	73.4 (67.6–78.6)	71.9	96.5
		No	7	193				
Trimethoprim-sulfamethoxazole	*dfr* plus *sul*	Yes	163	4	94.2 (89.6–97.2)	98.6 (96.3–99.6)	97.6	96.5
		No	10	273				
Chloramphenicol	*catA1*, *catB3*, *floR*	Yes	224	2	93.3 (89.4–96.1)	99.0 (96.6–99.9)	99.1	92.9
		No	16	208				

a Full resistance and intermediate resistance are included as nonsusceptible.

b Quinolone resistance-determining region (QRDR) consists of *gyrA*-83 plus *gyrA*-87 plus *parC*-80.

c Plasmid-mediated quinolone resistance genes (PMQR) include *qnrB6*, *qnrD1*, *qnrS1*, *qnrS2*, *oqx_AB*, *patA*, and *aac(6′)-Ib-cr*.

Quinolone resistance is mediated by mutations in the quinolone resistance-determining region (QRDR; *gyrA*-83, *gyrA*-87, and *parC*-80) and/or the acquisition of plasmid-mediated quinolone resistance genes (PMQR) (30). At least one QRDR mutation was detected in 38 isolates (8.4%) (Table S2), leading to nalidixic acid resistance. PMQR were present in around half the collection, with *qnrS1* identified in 47.8% of the isolates (215/450), followed by *aac(6*′*)-lb-cr*, *qnrS2*, *qnrB6*, *qnrD1*, *oqxAB*, and *patA*. The carriage of QRDR mutations and/or PMQR predicted nalidixic acid resistance with a sensitivity of 96.2% and specificity of 73.4%. Seven different QRDR mutations were identified; 11 isolates harbored triple mutations associated with resistance to ciprofloxacin (Table S2). These organisms included 10 *S*. Kentucky ST198 (*gyrA*-S83F, *gyrA*-D87N, and *parC*-S80I) and 1 *S*. Indiana ST17 (*gyrA*-S83F, *gyrA*-D87G, and *parC*-S80R). Additionally, ciprofloxacin resistance was associated with the presence of PMQR in either a single QRDR mutation (*n* = 7) or no mutation (*n* = 20) background (Table S2). The presence of QRDR mutations and/or PMQR could explain nonsusceptibility to ciprofloxacin with 90.8% sensitivity and 93.2% specificity.

The most commonly identified β-lactamases were *bla*_TEM-95_ (58.7%; 264/450) and *bla*_CTX-M-55_ (11.6%; 52/450). The presence of β-lactamases corresponded with resistance to different β-lactams, including ampicillin (97.2% sensitivity, 99.4% specificity) and 3rd-generation cephalosporins (91.2% sensitivity, 99.2% specificity). Resistance to the latter was associated with *bla*_CTX-M_ extended-spectrum β-lactamases (ESBLs) (present in 55/450 isolates). The carbapenemase *bla*_NDM-1_ was present in one *S*. Typhimurium ST34, but phenotypic testing found it susceptible to imipenem. Resistance to azithromycin has been reported previously in *Salmonella* and is chiefly attributed to the presence of the macrolide inactivation gene cluster (*mphA-mrx-mphR*) within a *Salmonella* genomic island (SARGI) (31). Herein, 58 NTS isolates carried the *mphA-mrx* construct, followed by other less common macrolide resistance genes such as *ermF*, *ermT*, *ermB*, and *mefB*. A genotype-phenotype comparison demonstrated that the presence of these genes was in high concordance with macrolide resistance (98.1% specificity).

Sulphonamide resistance was conferred by *sul2*, *sul3*, and *sul1*, which were present in 237, 116, and 33 of 450 NTS isolates, respectively. Resistance to trimethoprim could be explained by dihydrofolate reductase (*dfrA*) variants, with *dfrA12* (97/450) and *dfrA14* (69/450) being the most common. Genotypic prediction for trimethoprim-sulfamethoxazole resistance was calculated by combining the presence of both *sul* and *dfr* genes in each isolate, resulting in high sensitivity (94.2%) and specificity (98.6%). A large number of aminoglycoside resistance genes were prevalent, including the *aac(3)*, *aac(6′)*, *aph*, and *aad* families. Each of these is known to confer resistance to a distinct class of aminoglycosides (Table S1). We observed a high concordance in genotype-phenotype prediction for gentamicin (Table 3). However, in some instances, the presence of these genes did not translate to phenotypic resistance. For example, *aac(6*′*)-Iy* is chromosomally encoded and specific to *Salmonella*, but it is not usually transcribed due to the absence of an upstream promoter (32). This resulted in the observed genotype-phenotype mismatch in resistance to amikacin.

### Antimicrobial resistance differs between the major *Salmonella* sequence types.

**FIG 2:**
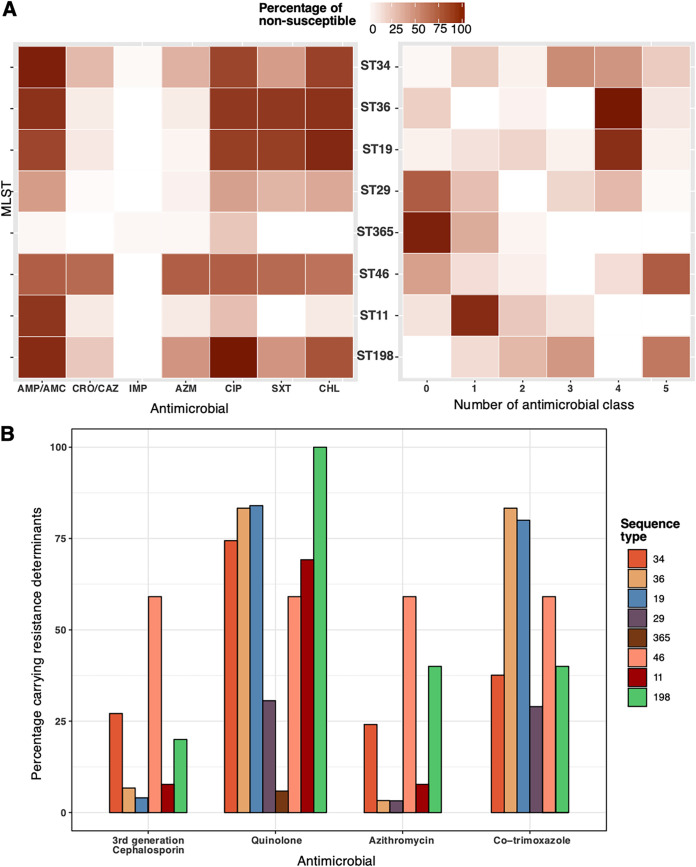
Antimicrobial resistance in major nontyphoidal *Salmonella* sequence types. (A) Heat map of antimicrobial resistance phenotype of the eight most common sequence types. The left panel displays the proportion of nonsusceptibility to 7 classes of tested antimicrobial agents, including ampicillin/amoxicillin-clavulanate (AMP/AMC), ceftriaxone-ceftazidime (CRO/CAZ), imipenem (IMP), azithromycin (AZM), ciprofloxacin (CIP); trimethoprim-sulfamethoxazole (SXT), and chloramphenicol (CHL). The right panel displays the proportion of nonsusceptibility to the number of tested antimicrobial classes, including β-lactam (AMP, AMC, CRO, and CAZ), IMP, AZM, CIP, SXT, and CHL. Isolates were classified as nonsusceptible to an antimicrobial class if they were nonsusceptible to any agent of that class. The color intensity in a cell is proportional to the percentage of nonsusceptible NTS isolates to the tested antimicrobial class. The STs are arranged in decreasing order of prevalence (from top to bottom). (B) The prevalence of antimicrobial resistance determinants in the eight most common sequence types. These determinants are classified by antimicrobial classes, including 3rd-generation cephalosporin (*bla*_CTX-M-14_, *bla*_CTX-M-15_, and *bla*_CTX-M-55_), quinolone [*qnrS1*, *qnrS2*, *qnrB6*, *qnrD1*, *oqxAB*, *patA*, *aac(6′)-Ib-cr*, and QRDR mutations], azithromycin (*ermB*, *ermF*, *ermT*, *mefB*, and *mphA*), and cotrimoxazole (*sul1*, *sul2*, and *sul3 plus* + *dfrA1*, *dfrA12*, *dfrA14*, *dfrA17*, and *dfrA5*). Each bar graph denotes the proportion of isolates carrying any of these determinants, classified by antimicrobial class and sequence type.

We proceeded to compare the AMR profiles of the eight most common STs (Fig. 2A) in order to gain insights into potential broader treatment strategies. There was significant variation in the AMR profile of these major STs. Nearly 80% (149/188) of *S*. Typhimurium were MDR, with the majority of the biphasic ST19 and ST36 exhibiting nonsusceptibility to four antimicrobial classes (ampicillin/amoxicillin-clavulanate, chloramphenicol, ciprofloxacin, and cotrimoxazole). This profile was due to the acquisition of the corresponding *bla*_TEM-95_, *floR*, *qnrS1*, and *dfrA12-sul* (Fig. 2B). *S*. Typhimurium ST34 displayed a more variable AMR profile, with 33.8%, 30.8%, and 14.3% of these organisms nonsusceptible to three, four, and five antimicrobial classes, respectively. Noticeably, the presence of *bla*_CTX-M_ or *mphA-mrx* was more frequent in ST34 (∼25%), resulting in a marked increase in the resistance to ceftriaxone and azithromycin, respectively. A high proportion of MDR was also evident in *S*. Newport ST46 (59.1%; 13/22) and *S*. Kentucky ST198 (70%; 7/10). Half of ST46 (*n* = 11) were nonsusceptible to all five classes of tested antimicrobials, owing to the presence of *bla*_TEM-95_, *bla*_CTX-M-55_, *aac(3)-IIa*, *aph(6′)-Id*, *aph(3′)-Ia*, *aadA22/24*, *mphA-mrx*, *qnrS1*, *dfrA14-sul3*, *floR*, *linG*, and *arr2/arr3* (Fig. 2). *S*. Stanley ST29 and *S*. Weltevreden ST365 were generally susceptible to all classes of antimicrobials. No AMR determinants were detected in *S*. Weltevreden ST365, except for two isolates carrying the chromosomally encoded *aac(6′)-Iy* and *qnrS1*.

To explore some additional features of the major NTS genotypes, we sought to compare the demographic and clinical data associated within the six most prevalent STs. Age was the only variable with a significant difference among these STs (Kruskal-Wallis test; adjusted *P = *0.0057); the median age of children infected with *S*. Weltevreden ST365 was 4.52 months (IQR, 3.48 to 9.4), the lowest among the compared STs. Disease severity factors, such as duration of hospitalization, frequency of diarrhea, and blood neutrophil count, were not significantly different between these six STs (Fig. S1). However, we identified two cases in which imipenem was given as the last resort after unsuccessful recovery with other antimicrobials, and both infections were caused by MDR *S*. Newport ST46. The first case was a 7.5-month-old male who was treated initially with macrolides and then fluoroquinolones. Imipenem was later administered, and the patient was hospitalized for 19 days. The second case was a 3-month-old male who was treated initially with intravenous ceftriaxone and hospitalized for 9 days.

### Resistance to first-line antimicrobials correlates with disease severity.

Treatment regimens were recorded for all enrolled patients, which included ORS, intravenous rehydration, zinc, probiotics, and antimicrobials. More than 90% of patients were administered with ORS and zinc supplementation (Table 1). Antimicrobials were also frequently prescribed, with 92.4% (423/450) of patients receiving empirical antimicrobial treatment following admission to the hospital and prior to obtaining a microbiological susceptibility test result. Most of the infected children recovered or had their symptoms improved after 3 days of enrollment; the children were discharged from the hospital after a median of 5 days (IQR, 3 to 7 days) (Table 1). Fluoroquinolones were most commonly used for primary treatment (299/423; 70.7%), followed by 3rd-generation cephalosporins (85/423) and macrolides (32/423). These initial treatments may be changed to a different (secondary or tertiary) antimicrobial, dependent on the patient’s clinical progression. Such changes occurred in 12.3%, 11.8%, and 25% of the corresponding fluoroquinolone, 3rd-generation cephalosporin, and macrolide initial treatments. For all three scenarios, a change in antimicrobial therapy was significantly associated with a prolonged hospital stay (Wilcoxon signed-ranked test; *P < *0.05), possibly reflecting the patients’ worsening illness or as required for parenterally administered antimicrobial (i.e., ceftriaxone).

**FIG 3:**
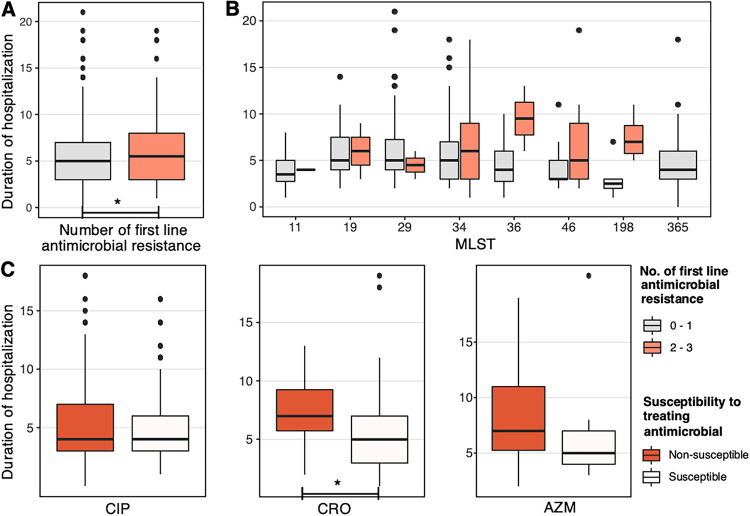
Resistance to first-line antimicrobials correlated with clinical severity. (A and B) Hospitalization duration (in days) of all 450 children infected with nontyphoidal *Salmonella*, classified by the number of first- and second-line antimicrobials (ciprofloxacin, ceftriaxone, azithromycin) to which the pathogen was phenotypically nonsusceptible (A), further classified by the eight most common sequence types (B). (C) Hospitalization duration of NTS infected children who received initial treatment of fluoroquinolone (CIP), 3rd-generation cephalosporin (CRO), or macrolide (AZM), stratified by the pathogen’s nonsusceptibility to the corresponding treating agent (ciprofloxacin, ceftriaxone, or azithromycin). The asterisk indicates statistical significance in the pairwise comparison (Wilcoxon signed-rank test, *P* < 0.05).

We have previously reported that the NTS MDR status was not associated with hospitalization duration or clinical outcome (13). As salmonellosis is frequently treated with either fluoroquinolones, 3rd-generation cephalosporins, or macrolides, we stratified the NTS cases by resistance to these antimicrobials only. For all patients, cases caused by NTS nonsusceptible to more than one of these drug classes had a small but significantly longer hospitalization (pairwise mean difference, 0.91 day; Wilcoxon signed-rank test; *P = *0.04) (Fig. 3A). This effect was observed throughout the major NTS STs (Fig. 3B). We next sought to understand how the nonsusceptibility to the treating agent influences disease recovery (Fig. 3C). For patients receiving initial fluoroquinolone treatment, nonsusceptibility to ciprofloxacin was not associated with a difference in hospitalization time. However, nonsusceptibility to ceftriaxone was significantly associated with a longer hospital stay in children treated with 3rd-generation cephalosporins (Wilcoxon signed-rank test; *P = *0.028). This effect was also observed with macrolide treatment but was not significantly different. These observations were not confounded by age, as there was no significant difference in the age of patients receiving these different antimicrobial treatments (Kruskal-Wallis test; *P > *0.05). Additionally, we performed the same statistical analyses on the original full data set (478 NTS diarrhea cases), which produced analogous results, demonstrating the robustness of our observations despite the sample size reduction.

## DISCUSSION

Our study combined a wealth of clinical, microbiological, and genomic data to uniquely detail the characteristics of NTS infections in southern Vietnam. We found that *Salmonella* gastroenteritis exhibits clear seasonality, with the prevalence of disease increasing in May through September. This pattern has also been recapitulated in NTS surveillance studies in Guangdong, China (33), and Bangkok, Thailand (34). The incidence rate of dysentery was previously found to be significantly higher between May and October in Vietnam, particularly for the southeast region where our study was conducted (35). This period coincides with the rainy season, with higher precipitation and humidity, which could grant higher survival and transmissibility of bacterial pathogens in the environment. This stands in contrast with the seasonal pattern of viral diarrhea, of which the burden is highest in January to March in southern Vietnam (12, 36).

We observed a great diversity of circulating *Salmonella*, with the monophasic *S*. Typhimurium ST34 dominating the NTS epidemiological landscape. This observation resonates with recent findings elsewhere in Asia, including China (33). The monophasic ST34 is strongly associated with swine food production (37) and has risen to prominence in Europe and globally during the last 2 decades (38). Genomic and phenotypic investigations have suggested that this variant is ecologically successful due to its extensive repertoire of antimicrobial and heavy metal resistance genes (29, 39). Indeed, the ST34 isolated from our study show elevated resistance to several antimicrobials of first-line treatments, especially ceftriaxone and azithromycin. We also recently discovered a novel biphasic, MDR ST34 clone causing invasive diseases in HIV-infected patients in Vietnam (8, 40). It is therefore of epidemiological interest how this invasive clone is genetically related to the diarrheagenic ST34 described herein, and such genomic analysis is being conducted. Other major STs, such as *S*. Weltevreden ST365, *S*. Stanley ST29, and *S*. Kentucky ST198, have been frequently reported in Asia (33, 41). Similar to previous reports, the majority of *S*. Stanley (ST29) and *S*. Weltevreden (ST365) were susceptible to all tested antimicrobials (18, 33). In contrast, all *S*. Kentucky (ST198) isolated displayed resistance to ciprofloxacin and are likely to belong to the Asian expansion of the internationally disseminating ST198 (42). While *S*. Enteritidis is predominant in previous surveillances in Greece (43), China (44), Tunisia (45), and the United States and is emerging in Australia, we only documented 17 cases attributed to this serovar. This discrepancy is explicable due to the younger age of our cohort (median of 9 months old), as *S*. Enteritidis has been mainly reported in infections of children >3 years old (33).

More than half of the isolated NTS were classified as MDR, with particular high nonsusceptibility occurrence to commonly prescribed antimicrobials, such as ciprofloxacin. The major genetic mechanism for resistance was the widespread carriage of *qnrS1*, which could be maintained by the sustained use of the antimicrobial locally. Nevertheless, this does not preclude the importation and local propagation of a pandemic-resistant clone, as likely in the case of ciprofloxacin-resistant ST198 *S*. Kentucky, or as proven previously for Shigella sonnei in Vietnam (46). The high concordance between AMR genotype and phenotype was observed in our collection, with sensitivity and specificity surpassing 90% for all classes of antimicrobials tested, except for imipenem, nalidixic acid, and azithromycin. Such high genotype-phenotype agreement has been noted in the *Salmonella* collection housed in Public Health England, United Kingdom (47). However, this study did not report testing for the aforementioned three antimicrobials. The low sensitivity in azithromycin nonsusceptibility prediction indicates that other resistance mechanisms, such as the recently determined mutations in *acrB* (48), remained uncharacterized. Alternatively, the low specificity in the case of nalidixic acid is likely due to the nonfunctionality or insufficient expression of the genetic determinants. These discrepancies warrant further research to improve the accuracy in inferring AMR phenotypes using sequencing data, which is applicable in public health surveillances or culture-independent multiplex molecular assay (49).

Differing *Salmonella* STs were not associated with the clinical outcome in our patient cohort. Clinical severity also did not differ among patients infected with different NTS serogroups in Thailand (34). However, two patients infected with MDR *S*. Newport ST46 had to be treated with the last-resort antimicrobial imipenem, showing that tailored antimicrobial treatment remains crucial in certain scenarios. *S*. Newport ST46 is infrequently described in Asia and has been linked to reservoir in reptiles (50). Its pan-resistance and epidemiological ambiguity require further investigation. Additionally, our analysis indicated that disease recovery may take longer if the NTS organism was nonsusceptible to the treating antimicrobial, particularly for ceftriaxone and likely azithromycin. Here, NTS infections most commonly occurred in the young age group, particularly children under 1 year old. These patients presented with many severe clinical symptoms, including high bloody fecal output, fever, and vomiting, suggesting the highly infectious and severe nature of this disease. Updated guidelines advise that antimicrobials should not be used routinely in NTS infections, except for the immunocompromised, the neonates, and the elderly (6, 9; https://www.who.int/news-room/fact-sheets/detail/salmonella-(non-typhoidal). However, antimicrobial treatment should be considered carefully to both benefit patients with moderate to severe symptoms and to limit the chances of developing resistance.

Our observational study was limited to the description of hospitalized cases with mucoid/bloody diarrhea; NTS was the most common pathogen confirmed by microbiological culturing. Therefore, other pathogens or cases of coinfection were not analyzed. Also, the burden of NTS causing milder or acute watery diarrhea was not accounted for. As we did not record detailed epidemiological data (i.e., diet, animal contact, and household cases), it was not possible to identify the possible source of infection. NTS are widely distributed in humans, animals, and environmental reservoirs, so the transmission route may differ on a case-by-case basis. The infections reported in our study could be either associated with zoonotic transmissions (through the food chain/contact with animals) or via sustained human-to-human transmissions distinct from the animal NTS reservoirs (51, 52). For instance, NTS isolated from asymptomatic close contacts have been shown to be closely related to ones causing invasive diseases in children in Africa (53). Our study relied on molecular serotyping (SISTR), without conducting phenotypic serotyping due to the shortage of trained personnel and dedicated resources. This approach has become attractive for its high-throughput and accurate performance and produces high concordance between genotyping and phenotypic serotyping in Shigella flexneri (54). Recently, the web-based application EnteroBase has independently employed serotype prediction from *Salmonella* genomes, which largely produces congruent results with the SISTR outputs (55).

In conclusion, NTS has become the most common bacterial pathogen detected in children hospitalized with bloody or mucoid diarrheal diseases in HCMC. *S*. Typhimurium was the predominant serovar in this setting and was associated with a variety of AMR genes leading to a high rate of MDR in this serovar. We observed an increasing trend of AMR, especially against the first- and second-line antimicrobial classes recommended by the WHO, i.e., ciprofloxacin, ceftriaxone, and azithromycin. Multiresistance could lead to prolonged hospitalized duration and the difficulty of choosing empirical antimicrobials for dysenteric diarrhea. Our work shows that WGS is a powerful method to characterize the serovar diversity and AMR profiles in NTS. A phylogenetic investigation of these NTS isolates in the context of global and/or invasive collections is the next step to better understand transmission dynamics and the evolutionary processes underpinning the circulation of these organisms.

## Supplementary Material

Supplemental file 1
