# Supplementary material for: Genomic Serotyping, Clinical Manifestations, and Antimicrobial Resistance of Nontyphoidal Salmonella Gastroenteritis in Hospitalized Children in Ho Chi Minh City, Vietnam
Source: J Clin Microbiol. 2020 Nov 18;58(12):e01465-20. doi: 10.1128/JCM.01465-20 (PMC7685882; doi:10.1128/JCM.01465-20)
Supplement: Supplemental file 1 [file JCM.01465-20-s0001.pdf]

Table S1. Prevalence of antimicrobial genes (AMR genes) among non-typhoidal *Salmonella* isolates from children hospitalised with diarrhoeal diseases in Ho Chi Minh City (N=450)

Classes	Antimicrobial agents	AMR genes	Count
**β-lactams**	**Ampicillin**	*blaTEM-95*	264
		blaOXA-1	6
		blaCARB-3	2
		blaCTX-M-15 + *blaSHV-66*	1
	**Cephalosporins III**	*blaCTX-M-55*	52
		blaCMY-2/ blaCMY-42	5
		blaCTX-M-14	2
	**Imipenem**	*blaNDM-1*	1
**Aminoglycosides**	**Gentamicin**	*aac(3)-IIa*	86
		aac(3)-IV	5
		aac(6)-IIa	1
	**Amikacin**	*aac(6)-Iy*	237
		aac(6)-Iaa	213
		aac(6)-Ib4	10
		aac(6)-Ib-cr	10
	**Streptomycin**	*aph(6)-Id* (*strB*)	186
		aadA2/ aadA3	132
		aadA12/ aadA15 / aadA17	130
		aadA22-24	115
		aadA8/ aadA25	13
		aadA7	10
		aadA16	4
		aadA5	2
	**Kanamycin**	*aph(3')-Ib* (*strA*)	168
		aph(3')-Ia	56
	**Hygromycin B**	*aph(4)-Ia*	5
**Macrolides**	**Azithromycin**	*mphA* + *mrx*	58
		ermF	7
		mphA	2
		ermT	2
		ermB	1
		mefB	1
**Quinolones**	**Ciprofloxacin/ Nalidixic acid**	*qnrS1*	215
		qnrS2	4
		qnrB6	3

		qnrD1	1
		oqx_AB	1
		patA	1
		aac(6)-Ib-cr	10
**Folate Pathway Inhibitors**	**Trimethoprim**	*dfrA12*	97
		dfrA14	69
		dfrA1	3
		dfrA17	2
		dfrA5	1
	**Sulfonamide**	*sul2*	237
		sul3	116
		sul1	33
**Phenicols**	**Chloramphenicol**	*floR*	225
		catB3	6
		catA1	1
**Tetracyclines**	**Tetracycline**	*tetA + tetR*	87
		tetA + tetR + tetD	1
**Lincosamides**	**Lincomycin**	*linG*	48
**Rifamycin**	**Rifampicin**	*arr2/ arr3*	50
**Cyclic peptides**	**Colistin**	*mcr_1*	6
**Antineoplastic**	**Bleomycin**	*ble*	1

Table S2. The prevalence of mutations in the quinolone resistance determining region (QRDR) in non-typhoidal *Salmonella* isolated from children hospitalized with diarrheal diseases and the association with fluoroquinolone resistance

***gyrA*_83**	***gyrA*_87**	***parC*_80**	**CIP[a]**	**Serovar**	**ST[b]**	**Count**	**Note**
**Y**	D	S	R	Saintpaul	50	6	all isolates carried *qnrS1*
			I	Give	516	5	4/6 carried *qnrS1*
			I	Emek	76	1	
**F**	**N**	**I**	R	Kentucky	198	10	4/10 isolates carried *qnrS1*
S	**N**	S	S	Enteritidis	11	8	
			R	Typhimurium (biphasic)	34	1	carried *oqxAB* and *aac(6)-Ib-cr*
**F**	D	S	I	Albany	292	2	
			I	Hadar	33	1	
S	**G**	S	I	Typhimurium (monophasic)	34	1	
			I	Indiana	17	1	
S	**Y**	S	I	Enteritidis	11	1	
**F**	**G**	**R**	R	Indiana	17	1	carried *aac(6)-Ib-cr*

[a] Inhibition zone diameter interpretive criteria for ciprofloxacin susceptibility according to CLSI standard[14] as followed: ≤20mm means full resistance (R), 21-30mm means intermediate (I) and ≥31mm means susceptible (S)

[b] Multilocus sequence typing

Amino acids letter code: tyrosine (Y), aspartic acid (D), serine (S), phenylalanine (F), asparagine (N), isoleucine (I), glycine (G), arginine (R)

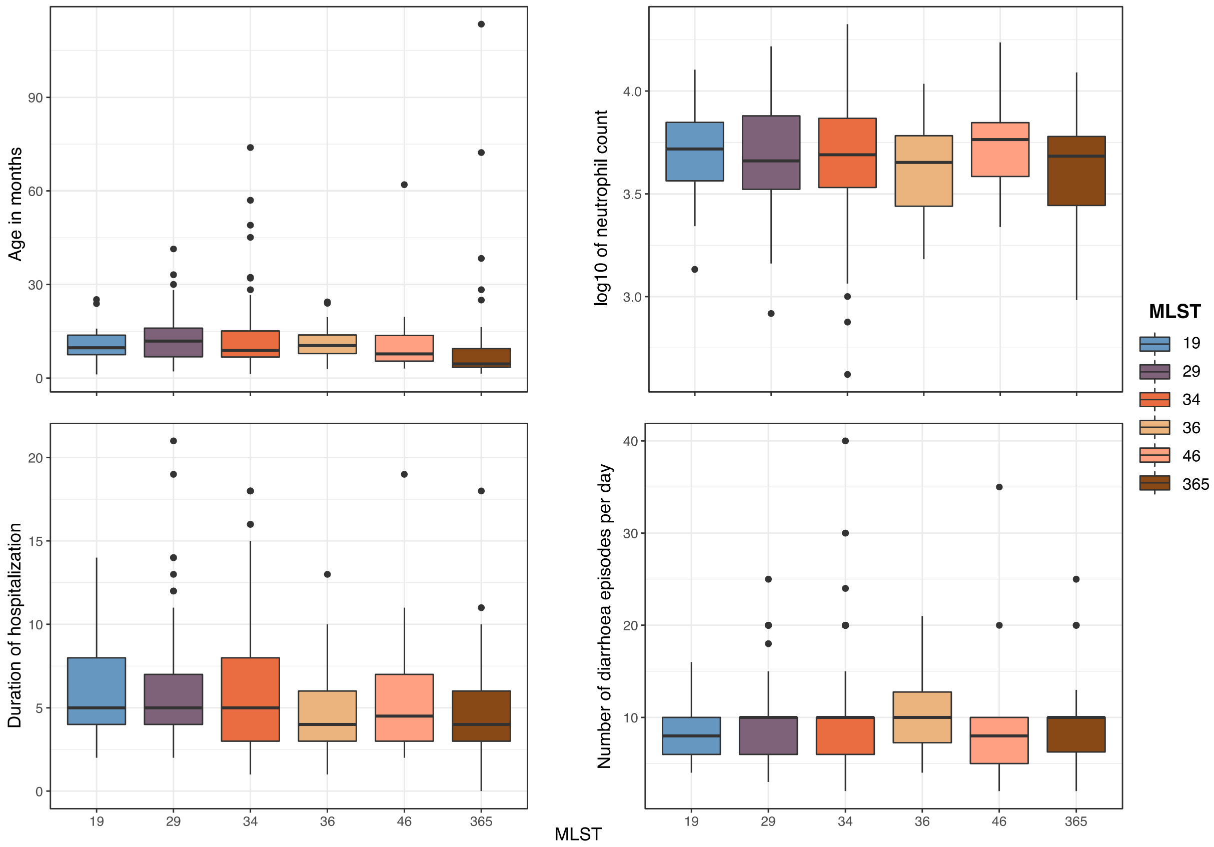

Figure S1. The demographic and clinical features of NTS disease

Comparison of demographic (age in months) and clinical (log10 of neutrophil count, duration of hospitalization, number of diarrhoea episodes per day) data among the six most common Non-typhoidal *Salmonella* sequence types recovered in this study.
